# Four Tick-Borne Microorganisms and Their Prevalence in *Hyalomma* Ticks Collected from Livestock in United Arab Emirates

**DOI:** 10.3390/pathogens10081005

**Published:** 2021-08-09

**Authors:** Nighat Perveen, Sabir Bin Muzaffar, Mohammad Ali Al-Deeb

**Affiliations:** Department of Biology, College of Science, United Arab Emirates University, Al Ain P.O. Box 15551, United Arab Emirates; 201790740@uaeu.ac.ae (N.P.); s_muzaffar@uaeu.ac.ae (S.B.M.)

**Keywords:** *Hyalomma dromedarii*, *Hyalomma anatolicum*, tick-borne pathogens, *Francisella*, *Rickettsia*, *Theileria annulata*, *Theileria ovis*, livestock

## Abstract

Ticks and associated tick-borne diseases in livestock remain a major threat to the health of animals and people worldwide. However, in the United Arab Emirates (UAE), very few studies have been conducted on tick-borne microorganisms thus far. The purpose of this cross-sectional DNA-based study was to assess the presence and prevalence of tick-borne *Francisella* sp., *Rickettsia* sp., and piroplasmids in ticks infesting livestock, and to estimate their infection rates. A total of 562 tick samples were collected from camels, cows, sheep, and goats in the Emirates of Abu Dhabi, Dubai, and Sharjah from 24 locations. DNA was extracted from ticks and PCR was conducted. We found that *Hyalomma dromedarii* ticks collected from camels had *Francisella* sp. (5.81%) and SFG *Rickettsia* (1.36%), which was 99% similar to *Candidatus* Rickettsia andeanae and uncultured *Rickettsia* sp. In addition, *Hyalomma anatolicum* ticks collected from cows were found to be positive for *Theileria annulata* (4.55%), whereas *H. anatolicum* collected from goats were positive for *Theileria ovis* (10%). The widespread abundance of *Francisella* of unknown pathogenicity and the presence of *Rickettsia* are a matter of concern. The discovery of *T. ovis* from relatively few samples from goats indicates the overall need for more surveillance. Increasing sampling efforts over a wider geographical range within the UAE could reveal the true extent of tick-borne diseases in livestock. Moreover, achieving successful tick-borne disease control requires more research and targeted studies evaluating the pathogenicity and infection rates of many microbial species.

## 1. Introduction

Ticks are hematophagous ectoparasites of a wide range of vertebrate hosts, including humans and animals, that play a significant role in the transmission cycles of various zoonotic diseases caused by viruses, bacteria, and protozoans [1,2]. Mixed tick-borne microorganism infections are common in nature and research and epidemiological investigations suggest that infections caused by mixed tick-borne microbiota can modulate their pathogenicity and disease burden in various hosts [3]. Nevertheless, the incidence of tick-borne infections is increasing in various parts of the world [4,5]. Crimean–Congo hemorrhagic fever (CCHF), rickettsioses, tularemia, anaplasmosis, ehrlichiosis, babesiosis and tropical theileriosis are the most common tick-borne diseases transmitted by the genus *Hyalomma* in the Middle East and North Africa region (MENA) [6]. The distribution of tick vector species and tick-borne pathogens are continuing to expand and overlap [4]. Multiple factors are involved in the increase in tick-borne diseases in the MENA region including expansion of tick geographic ranges due to wide-ranging livestock farming, import of animals from other geographic regions, an abundance of wildlife populations that support ticks lifecycles, and improved diagnostics and surveillance [6]. Thus, *Hyalomma* (Acari: Ixodidae) species which are widespread in the MENA region, Southern Europe, Central, Southern and South-Eastern Asia [6,7,8] pose a serious threat to human and animal health, and food security [6].

*Francisella tularensis* is the causative agent of a zoonotic disease referred to as tularemia, which can be fatal to humans and animals [9]. Transmission to humans occurs by direct contact, inhalation of aerosolized organisms or ingestion of contaminated material(s), or through bites of arthropods such as ticks and mosquitoes [10]. Tularemia occurs in the northern hemisphere and more often in Scandinavia, Russia, northern America, and Japan, however, tularemia has recently been reported from Europe with the highest annual incidence in Kosovo and Turkey and has become a significant re-emerging disease globally [9,10,11]. In addition, it has been reported from Middle East countries including Iran and Turkey [12]. Small mammals and arthropods, especially ticks (*Dermacentor*, *Amblyomma*, *Haemaphysalis* and *Ixodes*) play a vital role in the terrestrial cycle of *F. tularensis* [11,13]. Ticks are significant in the persistence of these bacteria in nature and transmit them to wild animals and livestock. Furthermore, ticks may serve as reservoirs and can carry bacteria in their bodies throughout their lives [11]. *Francisella* transmission in ticks can occur transtadially [14] as well as transovarially [15]. Several *Francisella* variants have been detected in ticks. In addition to *F. tularensis*, closely related bacteria broadly categorized as “*Francisella*-like endosymbionts” (FLE) may exist widely in ticks. These endosymbionts are assumed to be nonpathogenic to humans and may cause limited pathogenicity in small animals [16]. *Francisella*-like endosymbionts have been identified in tick species including *Dermacentor*, *Amblyomma*, *Ornithodoros*, *Ixodes*, and *Hyalomma* [17,18,19,20,21,22]. Moreover, they have been reported in the camel tick, *Hyalomma dromedarii* [19,23,24]. Overall, there is a need to determine the pathogenicity of these microbes widely categorized as FLE. 

Tick-borne rickettsioses are caused by members of the genus *Rickettsia*, which are obligate intracellular bacteria belonging to the spotted fever group (SFG) of the order Rickettsiales [25,26]. It is worth noting that the Mediterranean spotted fever caused by *Rickettsia conorii* is found in various Arab countries [6]. In addition, *Rickettsia* spp. have been reported previously in *H. dromedarii* ticks [27,28,29]. Assessment of the pathogenicity of *Rickettsia* species [30] has shown that species assumed to be nonpathogenic symbionts (e.g., *Rickettsia helvetica* and *Rickettsia slovaca*) [26] were actually pathogenic [30].

Piroplasmoses are widespread arthropod-borne infections of domestic and wild vertebrates that are caused by hemoprotozoan parasites of the phylum Apicomplexa that includes four genera: *Babesia*, *Theileria*, *Cytauxzoon*, and *Rangelia* [5]. Tropical theileriosis is a common tick-borne disease of ruminants including cattle, sheep, and goats, and a major threat to the cattle industry. Several species in the genus *Theileria* (Piroplasmorida: Theileriidae), for example, cause mortality and economic losses in cattle [31], and to a lesser extent in camels [32]. *Theileria* are obligate intracellular parasites transmitted by *Hyalomma* ticks. [32]. *Theileria annulata*, *T. parva*, *T. lestoquardi*, *T. luwenshuni*, *T. ovis*, and *T. uilenbergi* are mainly pathogenic to ruminants [33]. *Theileria* has been reported from most of the countries in the MENA region [6].

In the United Arab Emirates (UAE), camels were found to be infested with ticks throughout the year [34]; therefore, people who are in close contact with these animals such as farm workers, abattoir workers, veterinarians and researchers are at risk of being exposed to ticks and can have higher chances of getting infections with tick-borne pathogens. The widespread movement of animals in the livestock industry of the UAE suggests that microbes are likely to circulate in farms and periodically emerge as pathogens [6,27]. Thus, the present study was carried out to assess the presence and prevalence of tick-borne *Francisella* sp., *Rickettsia* sp., and piroplasmids in ticks infesting livestock using DNA-based methods, and to estimate their infection rates to better understand potential pathogens that threaten the livestock industry of UAE.

## 2. Results

### 2.1. Tick Identification

Ticks were identified as ixodids (Acari: Ixodidae). All ticks collected from camels were identified as *H. dromedarii*, while the ticks which were collected from cows, sheep and goats were identified as *H. anatolicum*, based on known morphological characteristics.

### 2.2. Detection of Francisella

**Figure 1:**
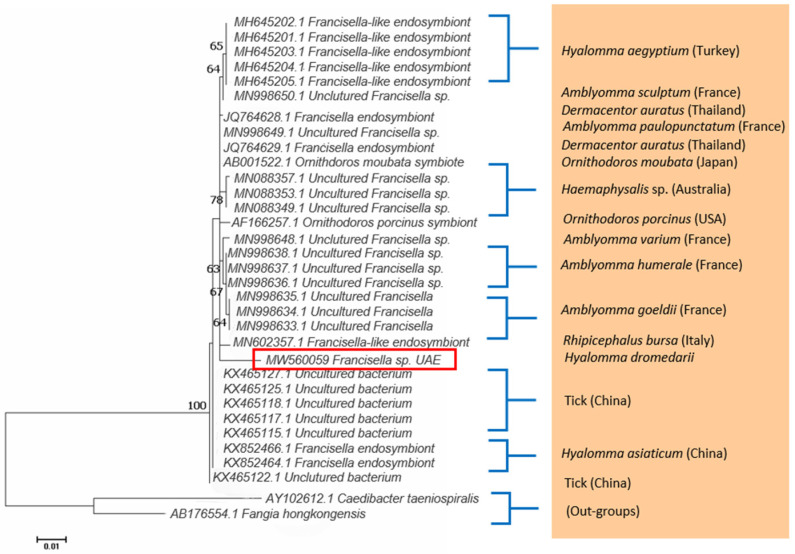
Maximum Likelihood tree based on the 16S rRNA gene showing the phylogenetic relationship of *Francisella* sp. detected in *H. dromedarii* from camels in Abu Dhabi, with reference sequences from the GenBank database. *Caedibacter taeniospiralis* and *Fangia hongkongensis* were used as out-groups. Column shows host names. The tree was generated with MEGA-X [35]. Red rectangle shows the accession number of the UAE sample.

*Francisella* sp. DNA was detected using PCR in *H. dromedarii* ticks from Abu Dhabi. Thirty tick samples had *Francisella* sp., which was very similar to a *Francisella*-like endosymbiont based on DNA similarity with the records in GenBank (Supplementary Table S1). A representative sequence was deposited in the GenBank (accession number MW560059). Samples were 98.59% identical to the *Francisella* sp. endosymbionts of *Amblyomma paulopunctatum* (MN998649.1), *Dermacentor auratus* (JQ764629.1), and *Ornithodoros moubata* (AB001522.1) (Supplementary Table S1). The phylogenetic tree (Figure 1) was constructed with *Francisella* sp. of this study and the sequences from the GenBank showing the highest similarity to it. The *Francisella* sp. DNA sequence of the present study formed a well-defined branch, which was supported by a significant bootstrap value. The overall inter-clade divergence was 0.49 ± 0.12 between the *Francisella* sp. DNA sequence (Abu Dhabi) of this study and the top ten matches from the GenBank. The pairwise genetic distance ranged from 1.44% (*Francisella* sp. UAE vs. uncultured *Francisella* sp. MN998649.1) to 1.68% (*Francisella* sp. UAE vs. uncultured *Francisella* sp. MN998636.1).

### 2.3. Detection of Rickettsia

**Figure 2:**
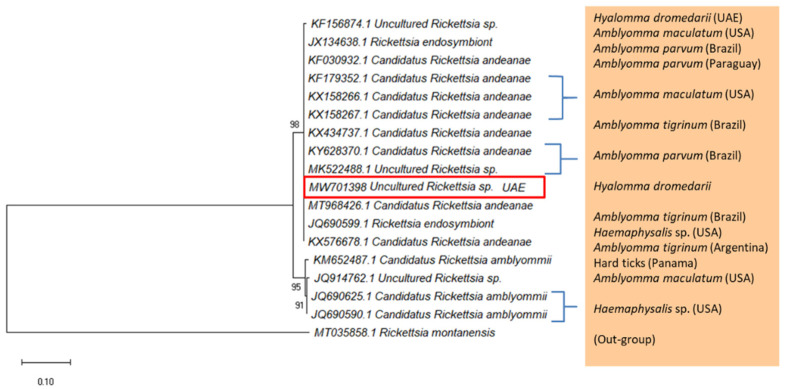
Maximum Likelihood tree based on the *ompA* gene showing the phylogenetic relationship of uncultured *Rickettsia* sp. detected in *H. dromedarii* from camels in Abu Dhabi, with reference sequences from the GenBank database. *Rickettsia montanensis* was used as an out-group. Column shows host names. The tree was generated with MEGA-X [35]. Red rectangle shows the accession number of the UAE sample.

Uncultured *Rickettsia* sp. DNA was only detected in *H. dromedarii* ticks collected from camels in Abu Dhabi. The 540 bp DNA fragment of the outer membrane protein *ompA* gene (Figure 2) was not detected in *H. dromedarii* and *H. anatolicum* collected from Dubai and Sharjah. Fragments were identified based on DNA sequence similarity with the records of the *ompA* gene from the GenBank (Supplementary Table S2). A representative sequence of uncultured *Rickettsia* sp. was deposited in the GenBank (accession number MW701398). This sequence was 99.8% identical to the *Candidatus* Rickettsia andeanae detected in *Amblyomma parvum* (KY628370.1) and *Amblyomma tigrinum* (KX434737.1) from Brazil, and *Amblyomma maculatum* from USA (KX158267.1). In addition, the sequence of the present study was 99.8% identical to uncultured *Rickettsia* sp. detected in *Amblyomma parvum* from Brazil (MK522488.1), and *H. dromedarii* from UAE (KF156874.1). The phylogenetic tree (Figure 2), which was constructed using highly similar GenBank sequences of *Rickettsia* sp. from this study showed that the UAE sample was in a cluster of *Cand.* R. andeanae and uncultured *Rickettsia* sp. Furthermore, the *Rickettsia* sp. was not found in ticks collected from other hosts such as cows, sheep, and goats.

### 2.4. Detection of Piroplasmids

**Figure 3:**
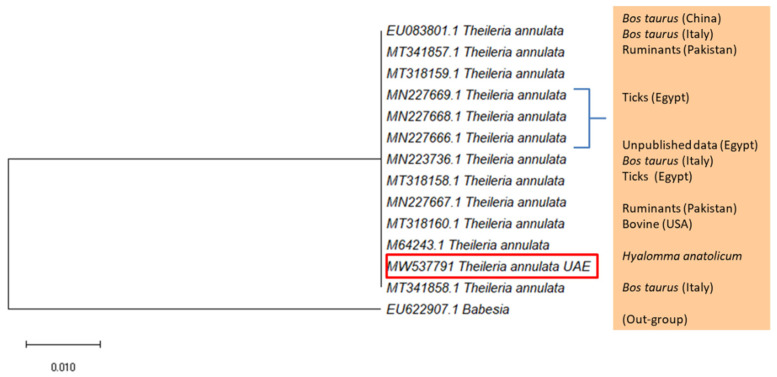
Maximum Likelihood tree based on the ssrRNA gene showing the phylogenetic relationship of *T. annulata* detected in *H. anatolicum* collected from cows in Sharjah, with reference sequences from the GenBank database. *Babesia major* was used as an out-group. Column shows host names. The tree was generated with MEGA-X [35]. Red rectangle shows the accession number of the UAE sample.

**Figure 4:**
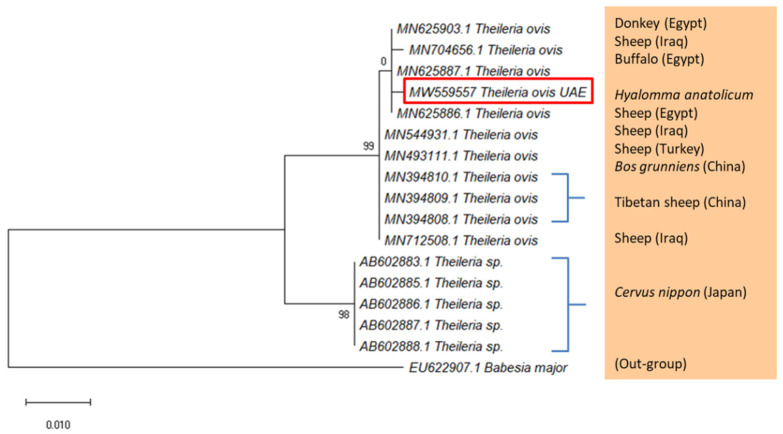
Maximum Likelihood tree based on the ssrRNA gene showing the phylogenetic relationship of *T. ovis* detected in *H. anatolicum* collected from goats in Sharjah, with reference sequences from the GenBank database. *Babesia major* was used as an out-group. Column shows host names. The tree was generated with MEGA-X [35]. Red rectangle shows the accession number of the UAE sample.

The DNA of *Theileria annulata* and *T. ovis* was detected using PCR in *H. anatolicum* ticks collected from cows and goats in Sharjah. Fragments of the ssrRNA gene were identified as *T. annulata* and *T. ovis* based on DNA similarity with the records in the GenBank (Supplementary Tables S3 and S4, respectively). The sequence of *T. annulata* was deposited in the GenBank with the accession number MW537791 and the one of *T. ovis* with MW559557. Samples of *T. annulata* were 99.62% identical to the *T. annulata* detected in cattle, *Bos taurus* (MT341858.1), ruminants (MT318160.1), and ticks (MN227669.1) (Supplementary Table S3). On the phylogenetic tree of *T. annulata* (Figure 3), which was constructed using GenBank sequences of high similarity to *T. annulata*, the UAE sequence appeared in a cluster of *T. annulata* samples detected from Italy, Pakistan, and Egypt. Similarly, sequences of *T. ovis* from the UAE were 99.81% identical to the *T. ovis* detected in cattle, *Bos grunniens* (MN394810.1) from China, Tibetan sheep (MN394809.1) from China, and sheep from Iraq (MN712508.1), and Egypt (MN625886.1). The phylogenetic tree (Figure 4) showed that the UAE sequence was in a cluster of *T. ovis* samples detected from Iraq and Egypt. Finally, piroplasmids were not detected in *H. dromedarii* and *H. anatolicum* from Abu Dhabi and Dubai.

### 2.5. PCR-Based Infection Rates of Tick-Borne Microbes

Tick-borne microbes were detected using PCR in 39 out of 562 DNA samples extracted from ticks, with overall infection rate of 6.94%. In Abu Dhabi, *H. dromedarii* collected from camels contained *Francisella* sp. (5.81%) and *Rickettsia* sp. (1.36%). However, these microbes were not detected in tick samples from Dubai and Sharjah. In Sharjah, *H. anatolicum* ticks collected from cows were found to be positive for *T. annulata* (4.55%). Moreover, *H. anatolicum* collected from goats in Sharjah were found to be positive for *T. ovis* (10%). No microbe was detected in the ticks collected from sheep in Abu Dhabi, Dubai, and Sharjah. Ticks collected from camels had a higher rate of infection compared with ticks collected from cows, sheep, and goats (Table 1, Supplementary Table S5). In addition, no microbe was detected in ticks collected from camels, cows, sheep and goats in Dubai. 

## 3. Discussion

Disease detection is the most important step in programs that safeguard human or animal health [6,36]. Early detection of pathogens is crucial in curtailing their spread and subsequently in reducing risk of exposure and possible outbreaks [6,36]. Our data revealed the presence of two bacterial and two piroplasmid species in local ticks infesting several animal hosts. *Hyalomma* ticks contained four microbes, namely, *Francisella* sp., *Rickettsia* sp., *T. annulata*, and *T. ovis*. We found that the highest infection (5.81%) was with *Francisella* sp. in the *H. dromedarii* ticks collected from camels in Abu Dhabi. These findings are comparable to infection rates of *Francisella* spp. (4.7%) in *H. dromedarii* from camels in Egypt [23]. However, the molecular identification of *Francisella* sp. in the present study aligned with *Francisella*–like endosymbionts rather than with any known pathogenic *Francisella* spp., which agrees with reports from Egypt [23]. In addition, the *Francisella* sp. of this study was closely related to *Francisella* endosymbiont recorded in *A. paulopunctatum* (MN998649.1) [37], *D. auratus* (JQ764629.1) and *O. moubata* (AB001522.1). It should be pointed out that previously the genus *Francisella* has been reported with a very high prevalence (99.1%) in *H. dromedarii* ticks from camels in the UAE [24]. Therefore, future studies in the UAE should use species-specific primers to determine whether *Francisella* sp. is pathogenic or a *Francisella*–like endosymbiont.

*Rickettsia* sp. in *H. dromedarii* ticks collected from camels in Abu Dhabi in our study was closely related to *Cand.* R. andeanae recorded in *Amblyomma* ticks from Brazil and the USA. In addition, it was also 99.8% identical to uncultured *Rickettsia* sp. detected previously in *Amblyomma* and *Hyalomma* ticks from Brazil and the UAE, respectively. Many *Rickettsia* species exist in ticks, although their pathogenicity has not been determined [38]. Since ticks may serve as vectors as well as reservoirs of rickettsiae in nature, this constitutes a risk factor for *Rickettsia* transmission in livestock and humans [38]. Spotted fever group (SFG) rickettsiae have at least 30 distinct genotypes in 15 species currently recognized as pathogens in humans [39,40]. Recently, *R. parkeri* (that causes spotted fever rickettsiosis in humans) and *Cand.* R. andeanae were reported widely in several tick species across wide geographic regions [40,41,42]. Although *Cand.* R. andeanae do not seem to cause human infections [43], the high prevalence of *Cand.* R. andeanae in ticks might interfere with the development of *R. parkeri* and limit its distribution [42]. Therefore, there is a need to better quantify the dynamics among various spotted fever group *Rickettsia* species within their tick hosts to determine how their interactions contribute towards the epidemiology of rickettsioses in human and animal hosts [43]. Furthermore, ticks are known to engage in symbiotic associations with at least 10 different genera of maternally inherited bacteria [44]. Ticks develop close interactions with beneficial symbionts that provide essential B vitamins and other co-factors required for survival and reproduction [37,44,45]. Coexistence of *Francisella* and *Rickettsia* in ticks on camels from Abu Dhabi in our study highlights the need to characterize the interactions between diverse microbes in ticks [24,37].

Theileriosis has a large economic impact at the global level due to losses in the livestock industry [46]. Better control measures like immunization with a live attenuated vaccine has been effectively used to control theileriosis [46]. Many *Theileria* species have been reported across the MENA region [6]. In this study, we found *Theileria* spp. in Sharjah only and in low prevalence. This may be due to differences in breeds of livestock, farming conditions and frequency of acaricide application amongst the different Emirates. *Theileria* *ovis* is reported here for the first time in *H. anatolicum* ticks from goats in the UAE. The genotype was identical to *T. ovis* in cattle and sheep from Iraq and Egypt, suggesting that our genotype could be a geographically widespread variant. *Theileria* *annulata* detected in cattle in this study was identical to previously identified genotypes from the UAE [27] and clustered with *T. annulata* from Italy, Pakistan and Egypt, again suggesting that the variant was widespread. The prevalence of *Theileria* in livestock from Oman [33] and Saudi Arabia is also comparable to our findings [47]. Furthermore, the highest prevalence of *Theileria* infections occur in *H. anatolicum* compared to *H. excavatum*, *H. scupense* and *H. marginatum*, suggesting that *H. anatolicum* may be the main vector of theileriosis [31]. Thus, the Arabian Peninsula could be a region where theileriosis may become endemic in the future. The presence of Malignant Ovine Theileriosis (MOT) in Oman indicates that mixed species infections are associated with pathogen density regulation (presumably through within-host interactions), resulting in lower mortality [3]. The role of mixed infections of *Theileria* pathogens in the epidemiology of ovine theileriosis is required to be investigated for a control strategy and improved clinical outcome [3].

Overall, the findings of the current study highlight that tick-vectored microorganisms continue to be detected repeatedly in the UAE because of the increasing livestock industry and associated tick vectors. Thus, there is a need for annual large-scale disease screening programs. Furthermore, it may be suggested that detailed investigations of the abundance and diversity of these piroplasm pathogens and their mixed infections in vector populations (ticks) need to be performed all over the UAE. In addition, continuous surveillance is imperative to maintain good health of livestock and for the early detection of disease catastrophes in ruminants for food security.

## 4. Materials and Methods

### 4.1. Ethics Statement

Tick collection was carried out in strict accordance with the experimental protocol approved by the Animal Research Ethics Committee of the UAE University (ethical approval# ERA_2019_5953). We confirm that all methods were carried out in accordance with relevant guidelines and regulations.

### 4.2. Study Area, Tick Collection and Identification

**Figure 5:**
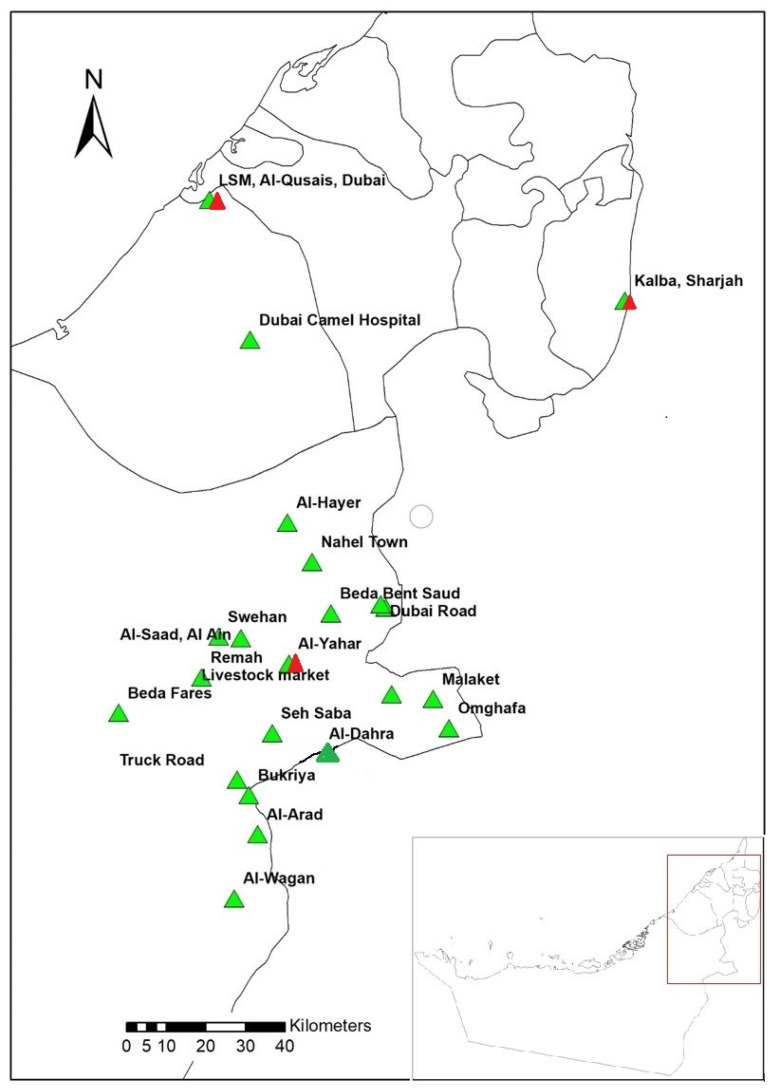
The study area from which tick samples were collected in the UAE; red triangles show the locations of sheep, cows and goats samples, while green triangles show the locations of camel samples except Livestock Market Al-Qusais, Dubai.

**Figure 6:**
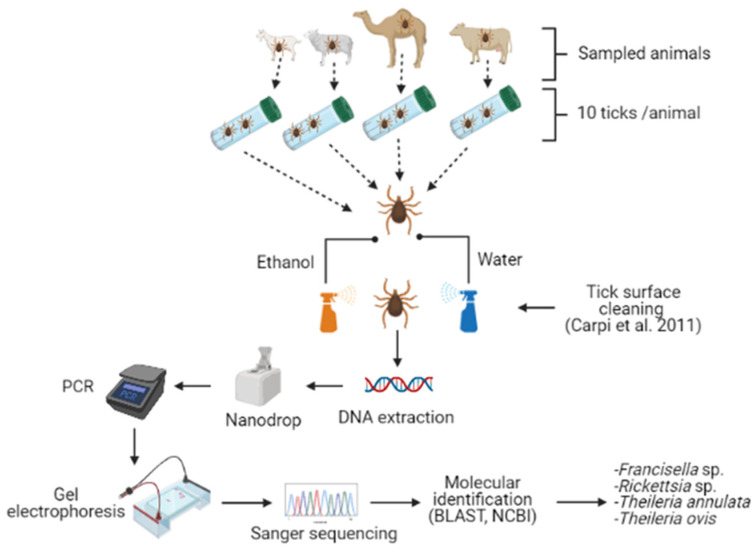
Experimental protocol. *Hyalomma* ticks (*n* = 10) were collected from each animal (camel, cow, goat, and sheep) from Abu Dhabi, Dubai, and Sharjah in the UAE. The figure was created with BioRender (https://biorender.com/).

This is a cross-sectional study in which tick collection was done from January 2019 to February 2020. A total 562 tick samples were collected from camels, cows, sheep and goats in the Emirates of Abu Dhabi, Dubai, and Sharjah from 24 locations (Supplementary Table S5; Figure 5). The largest number of ticks was collected from camels (516 samples), which represented the main animal in this study, whereas less samples were collected from sheep, goats, and cows (46 samples). Animals were selected randomly, and from each host 10 ticks were removed manually using a pair of forceps. Ticks were collected in 50 mL plastic tubes (Sterilin, Teddington, UK). All tick samples were placed in an icebox and transported to the Entomology Laboratory at the UAE University. Ticks were frozen at −20 °C until further processing. In Sharjah, a pool of ticks was created for each host (camels (3), sheep (25), goats (25), and cows (6)) (Supplementary Table S5). Similarly, a pool of ticks was created from the sheep (36) samples in Abu Dhabi. Further, a total of 15 cows (Australian origin) and 30 goats (Pakistani and Indian origin) were sampled in Abu Dhabi for tick collection; however, these animals were not infested with ticks (Supplementary Table S5). All ticks were identified at the species level on the basis of their morphology by using taxonomic keys [48,49] (Figure 6).

### 4.3. Genomic DNA Extraction

As for *H. dromedarii* ticks, DNA was extracted from individual ticks (partially engorged female). However, in the case of *H. anatolicum* ticks, DNA was extracted from a pool of 5 ticks (male) due to their small size. Further, partially engorged females of *H. anatolicum* were not available in all samples. Before the extraction, each tick was washed in 500 μL 70% ethanol followed by 500 μL sterile double-distilled H_2_O for five minutes to remove environmental contaminants attached to the tick body [50] and then dried for 10 min. Ticks were manually crushed using a plastic pellet pestle (Kimble, Fisher Scientific, Waltham, MA, USA) inside a sterile 1.5 mL micro-centrifuge tube by using liquid nitrogen. The DNeasy blood and tissue kit (Qiagen, Hilden, Germany) was used for tick genomic DNA extraction, following the protocol of the manufacturer. Extracted DNA samples were stored in a freezer at −80 °C.

### 4.4. Polymerase Chain Reaction

PCR tests were performed for *Francisella* sp. detection, with tick genomic DNA using an oligonucleotide primer pair (Table 2) [51] to amplify 1151 bp of the 16S rRNA gene. Detection of *Rickettsia* sp. was carried out by nested PCR of the *ompA* gene [52]. The amplification of a 590 bp fragment was obtained in the first PCR and the amplification of a 540 bp fragment was obtained in the second PCR using oligonucleotide primers (Table 2). The detection of *T. annulata* and *T. ovis* was done by PCR tests with tick genomic DNA using the oligonucleotide primer pair amplifying 560 bp of the ssrRNA gene [53] (Table 2). For all the above-mentioned microorganisms, each PCR reaction was carried out in 25 μL volume containing 12.5 µL Taq PCR master mix (Qiagen, Hilden, Germany), 1.0 µL (10 pM) of each primer, 3.0 µL of genomic DNA, and 7.5 µL nuclease-free water. PCR amplifications were carried out in a Swift MaxPro thermo-cycler (ESCO, Singapore) according to cycle conditions given in Table 2. Every PCR included a negative control (no template DNA) to detect any contamination. In addition, a positive control was used to indicate that the primers were properly annealing to the target region on the template DNA. In every PCR, we used filter tips and separate 0.2 mL tubes, rather than a PCR 96-well plate, to avoid aerosol cross-contamination between samples. In addition, PCR reaction tubes of the positive controls were prepared in a separate laboratory, to avoid any chance of contamination.

### 4.5. Agarose Gel Electrophoresis and Amplicon Purification

Products of PCR reactions were visualized using gel electrophoresis on 1.5% agarose gel, stained by ethidium bromide. The bands on the gel were visualized and photographed using a gel documentation system (Major Science, Taipei, Taiwan). Amplicons of the positive samples which produced the expected band size were purified using a PCR purification kit (Qiagen, Hilden, Germany) following the manufacturer’s protocol and saved for DNA sequencing.

### 4.6. DNA Sequencing, Phylogenetic Analysis, and Microorganism Identification

Purified PCR products were sequenced (Sanger sequencing) at the Biology Department sequencing unit, UAE University. Microorganisms were identified based on sequence analysis using the NCBI BLAST (http://blast.ncbi.nlm.nih.gov/Blast.cgi) analysis tool in the GenBank database (accessed on 13 January 2021) (Figure 6). Sequences were submitted in GenBank and received accession numbers (MW537791, MW559557, MW560059, and MW701398). The DNA sequences of this study were compared with known sequences listed in the GenBank nucleotide sequence databases. The obtained sequences were aligned using the MUSCLE program and the phylogenetic trees were constructed through the Maximum Likelihood approach using Kimura 2-parameter method and bootstrap analyses with 1000 replicates in MEGA X 10.0.5 software [35]. In each phylogenetic analysis, we chose the most suitable substitution model based on the lowest Bayesian Information Criterion scores (BIC). Consequently, after the DNA-based molecular identification of the four microorganisms, the infection rate of each microorganism was calculated from the number of samples from each host.

## 5. Conclusions

In this study, the main finding was that *H. dromedarii* ticks collected from camels had *Francisella* sp. (5.81%) and *Rickettsia* sp. (1.36%), whereas *H. anatolicum* ticks collected from cows were found to be positive for *T. annulata* (4.55%). Moreover, *H. anatolicum* collected from goats were positive for *T. ovis* (10%). Overall, more research is needed to better understand the microorganisms associated with *H. dromedarii* and *H. anatolicum* ticks. Moreover, the present study underscores the strong need to implement large-scale disease detection studies in livestock in the UAE.

## Figures and Tables

**Table 1:** Infection rate of bacteria and piroplasmids in ticks collected from livestock in Abu Dhabi, Dubai, and Sharjah, UAE.

Host	Tick Species	Number of Animals	Number of Samples	Number of Positive Samples (Total Infection Rate)			
				*Francisella* sp.	*Rickettsia* sp.	*T. annulata*	*T. ovis*
Camel	*H. dromedarii*	518	516	30 (5.81)	7 (1.36)	0 (0)	0 (0)
Sheep *	*H. anatolicum*	70	14	0 (0)	0 (0)	0 (0)	0 (0)
Goat *	*H. anatolicum*	34	10	0 (0)	0 (0)	0 (0)	1 (10)
Cow *	*H. anatolicum*	26	22	0 (0)	0 (0)	1 (4.55)	0 (0)
Total		648	562	30	7	1	1

* ticks were pooled into given numbers of samples.

**Table 2:** Primers and cycle conditions used to amplify gene fragments.

Pathogen	Target Gene	Primer	Sequence (5′–3′)	Cycle Conditions	Amplicon Size (bp)	Reference
*Francisella* sp.	16S rRNA	Fr153F0.1 Fr1281R0.1	GCCCATTTGAGGGGGATACC GGACTAAGAGTACCTTTTTGAGT	95 °C 4 min 40 cycles: 94 °C 30 s 60 °C 45 s 72 °C 60 s 72 °C 20 min	1151	[51]
*Rickettsia* sp.	*ompA*	RR 190-70 (1st PCR) RR 190-701(1st PCR)	ATGGCGAATATTTCTCCAAAA GTTCCGTTAATGGCAGCATCT	94 °C 1 min 35 cycles: 94 °C 30 s 50 °C 1 min 68 °C 4 min 72 °C 20 min	590	[52]
		190-FN1 (nested) 190-RN1 (nested)	AAGCAATACAACAAGGTC TGACAGTTATTATACCTC		540	
*Theileria* sp.	ssrRNA	Pirop-F Pirop-F	GTCTTGTAATTGGAATGATGG CCAAAGACTTTGATTTCTCTC	94 °C 2 min 35 cycles: 94 °C 30 s 50 °C 30 s 72 °C 60 s 72 °C 7 min	560	[53]

## Data Availability

Data is contained within the article or Supplementary Material.

## Supplementary Materials

The following are available online at https://www.mdpi.com/article/10.3390/pathogens10081005/s1, Table S1: Molecular identification of *Francisella* sp. endosymbiont isolated from *H. dromedarii* collected from camels in Abu Dhabi, UAE based on DNA similarity between 16S rRNA gene and GenBank species using NCBI BLAST, Table S2: Molecular identification of Uncultured *Rickettsia* sp. isolated from *H. dromedarii* collected from camels in Abu Dhabi, UAE based on DNA similarity between ompA gene and GenBank species using NCBI BLAST, Table S3: Molecular identification of *T. annulata* isolated from *H. anatolicum* collected from cows in Sharjah, UAE based on DNA similarity between ssrRNA gene and GenBank species using NCBI BLAST, Table S4: Molecular identification of *T. ovis* isolated from *H. anatolicum* collected from goats in Sharjah, UAE based on DNA similarity between ssrRNA gene and GenBank species using NCBI BLAST, Table S5: Prevalence of microbes in *Hyalomma* ticks in UAE.
