# Supplementary material for: Four Tick-Borne Microorganisms and Their Prevalence in Hyalomma Ticks Collected from Livestock in United Arab Emirates
Source: Pathogens. 2021 Aug 9;10(8):1005. doi: 10.3390/pathogens10081005 (PMC8398371; doi:10.3390/pathogens10081005)
Supplement: Supplementary file 1 [file pathogens-10-01005-s001.zip › pathogens-1260599-supplementary.pdf]

Table S1. Molecular identification of *Francisella* sp. endosymbiont isolated from *H. dromedarii* collected from camels in Abu Dhabi, UAE based on DNA similarity between 16S rRNA gene and GenBank species using NCBI BLAST.

**Best Match Species**	**Accession Number**	**Sequence Identity %**	**Sequence Coverage %**	***E*-value [a]**	**Host**	**Country**
Uncultured *Francisella* sp. clone FraApaulo	MN998649.1	98.59	100	0.0	*Amblyomma paulopunctatum*	France
Francisella endosymbiont	JQ764629.1	98.59	100	0.0	*Dermacentor auratus*	Thailand
Ornithodoros moubata symbiont	AB001522.1	98.59	100	0.0	*Ornithodoros moubata*	Japan
Uncultured *Francisella* sp. clone FraAscul	MN998650.1	98.47	100	0.0	*Amblyomma sculptum*	France
Francisella endosymbiont	JQ764628.1	98.47	100	0.0	*Dermacentor auratus*	Thailand
Uncultured *Francisella* sp. clone FraAhume3	MN998638.1	98.35	100	0.0	*Amblyomma humerale*	France
Uncultured *Francisella* sp. clone FraAhume2	MN998637.1	98.35	100	0.0	*Amblyomma humerale*	France
Uncultured *Francisella* sp. clone FraAhume1	MN998636.1	98.35	100	0.0	*Amblyomma humerale*	France
Uncultured *Francisella* sp. clone 627HBF ZOTU 13a	MN088353.1	98.35	100	0.0	*Haemaphysalis bancrofti*	Australia
Uncultured *Francisella* sp. clone 297HBF ZOTU 13a	MN088349.1	98.35	100	0.0	*Haemaphysalis bancrofti*	Australia
Francisella endosymbiont of *Ornitnodoros porcinus*	AF166257.1	98.35	100	0.0	*Ornitnodoros porcinus*	USA
Uncultured bacterium clone he_23	KX465127.1	98.35	100	0.0	Tick	China
Uncultured bacterium clone he_17	KX465125.1	98.35	100	0.0	Tick	China
Uncultured bacterium clone he_6	KX465118.1	98.35	100	0.0	Tick	China
Uncultured bacterium clone he_5	KX465117.1	98.35	100	0.0	Tick	China
Uncultured bacterium clone he_1	KX465115.1	98.35	100	0.0	Tick	China
Francisella endosymbiont isolate XJ-S3	KX852466.1	98.35	100	0.0	*Hyalomma asiaticum*	China
Francisella endosymbiont isolate XJ-S1	KX852464.1	98.35	100	0.0	*Hyalomma asiaticum*	China
Uncultured *Francisella* sp.	MN998635.1	98.24	100	0.0	*Amblyomma goeldii*	France
Uncultured *Francisella* sp.	MN998634.1	98.24	100	0.0	*Amblyomma goeldii*	France
Uncultured *Francisella* sp.	MN998633.1	98.24	100	0.0	*Amblyomma goeldii*	France
Uncultured *Francisella* sp. clone FraAvari	MN998648.1	98.24	100	0.0	*Amblyomma varium*	France
Uncultured *Francisella* sp. clone 297HBF ZOTU 13a	MN088357.1	98.24	100	0.0	*Haemaphysalis bancrofti*	Australia
Francisella endosymbiont of *Hyalomm marginatum*	AF166257.1	98.24	100	0.0	*Rhipicephalus bursa*	Italy
Francisella-like endosymbiont	MH645205.1	98.24	100	0.0	*Hyalomma aegyptium*	Turkey
Francisella-like endosymbiont	MH645204.1	98.24	100	0.0	*Hyalomma aegyptium*	Turkey
Francisella-like endosymbiont	MH645203.1	98.24	100	0.0	*Hyalomma aegyptium*	Turkey
Francisella-like endosymbiont	MH645202.1	98.24	100	0.0	*Hyalomma aegyptium*	Turkey
Francisella-like endosymbiont	MH645201.1	98.24	100	0.0	*Hyalomma aegyptium*	Turkey

[a] The typical threshold for a good *E*-value from a BLAST search is 10^{-5} or lower.

Table S2. Molecular identification of Uncultured *Rickettsia* sp. isolated from *H. dromedarii* collected from camels in Abu Dhabi, UAE based on DNA similarity between *omp*A gene and GenBank species using NCBI BLAST.

**Best Match Species**	**Accession Number**	**Sequence Identity %**	**Sequence Coverage %**	***E*-value[a]**	**Host**	**Country**
Uncultured *Rickettsia* sp. clone C269_18	MK522488.1	99.80	100	0.0	*Amblyomma parvum*	Brazil
Candidatus Rickettsia andeanae clone Caxias	KY628370.1	99.80	100	0.0	*Amblyomma parvum*	Brazil
Candidatus Rickettsia andeanae haplotype BQ-RS	KX434737.1	99.80	100	0.0	*Amblyomma tigrinum*	Brazil
Candidatus Rickettsia andeanae clone 4	KX158267.1	99.80	100	0.0	*Amblyomma maculatum*	USA
Candidatus Rickettsia andeanae clone 3	KX158266.1	99.80	100	0.0	*Amblyomma maculatum*	USA
Candidatus Rickettsia andeanae isolate Agripino Enciso	KF179352.1	99.80	100	0.0	*Amblyomma parvum*	Paraguay
Candidatus Rickettsia andeanae isolate Ap	KF030932.1	99.80	100	0.0	*Amblyomma parvum*	Brazil
Uncultured *Rickettsia* sp. clone ALAIN-001-2011	KF156874.1	99.80	100	0.0	*Hyalomma dromedarii*	UAE
Rickettsia endosymbiont of *Amblyomma maculatum* strain SH_B4	JX134638.1	99.80	100	0.0	*Amblyomma maculatum*	USA
Candidatus Rickettsia andeanae isolate At2	MT968426.1	99.80	100	0.0	*Amblyomma tigrinum*	Brazil
Candidatus Rickettsia andeanae isolate At2	JQ690599.1	99.80	100	0.0	*Haemaphysalis* sp.	USA
Candidatus Rickettsia amblyommii isolate 61A	JQ690625.1	94.78	98	0.0	*Haemaphysalis* sp.	USA
Candidatus Rickettsia andeanae isolate G614	KX576678.1	99.76	83	0.0	*Amblyomma tigrinum*	Argentina
Candidatus Rickettsia amblyommii isolate GP4A	KM652487.1	94.27	99	0.0	Hard ticks	Panama
Candidatus Rickettsia amblyommii isolate 23B	JQ690590.1	94.76	97	0.0	*Haemaphysalis* sp.	USA
Uncultured *Rickettsia* sp. clone SH_MG7	JQ914762.1	94.08	99	0.0	*Amblyomma maculatum*	USA

Table S3. Molecular identification of *T. annulata* isolated from *H. anatolicum* collected from cows in Sharjah, UAE based on DNA similarity between ssrRNA gene and GenBank species using NCBI BLAST.

**Best Match Species**	**Accession Number**	**Sequence Identity %**	**Sequence Coverage %**	***E*-value[a]**	**Host**	**Country**
Theileria annulata	MT341858.1	99.62	99	0.0	*Bos taurus*	Italy
Theileria annulata	MT341857.1	99.62	99	0.0	*Bos taurus*	Italy
Theileria annulata isolate T178	MT318160.1	99.62	99	0.0	Ruminants	Pakistan
Theileria annulata isolate T79	MT318159.1	99.62	99	0.0	Ruminants	Pakistan
Theileria annulata isolate T33	MT318158.1	99.62	99	0.0	Ruminants	Pakistan
Theileria annulata isolate Ticks, No 46	MN227669.1	99.62	99	0.0	Ticks	Egypt
Theileria annulata isolate Ticks, No 45	MN227668.1	99.62	99	0.0	Ticks	Egypt
Theileria annulata isolate Ticks, No 44	MN227667.1	99.62	99	0.0	Ticks	Egypt
Theileria annulata isolate Ticks, No 24	MN227666.1	99.62	99	0.0	Ticks	Egypt
Theileria annulata isolate 355	MN223736.1	99.62	99	0.0	Unpublished data	Egypt
Theileria annulata clone 5-31	AY508465.1	99.62	99	0.0	Cattle	Turkey
Theileria annulata isolate Turkey 4	AY508464.1	99.62	99	0.0	Cattle	Turkey
Theileria annulata isolate Turkey 3	AY508463.1	99.62	99	0.0	Cattle	Turkey
Theileria annulata	EU083801.1	99.43	99	0.0	*Bos taurus*	China
Theileria annulata	M64243.1	99.43	99	0.0	Bovine	USA

Table S4. Molecular identification of *T. ovis* isolated from *H. anatolicum* collected from goats in Sharjah, UAE based on DNA similarity between ssrRNA gene and GenBank species using NCBI BLAST.

**Best Match Species**	**Accession Number**	**Sequence Identity %**	**Sequence Coverage %**	***E*-value[a]**	**Host**	**Country**
Theileria ovis isolate HBOY1	MN394810.1	99.81	99	0.0	*Bos grunniens*	China
Theileria ovis isolate HXTS1	MN394809.1	99.81	99	0.0	Tibetan sheep	China
Theileria ovis isolate HBTS1	MN394808.1	99.81	99	0.0	Tibetan sheep	China
Theileria ovis isolate SH. T1	MN712508.1	99.81	99	0.0	Sheep	Iraq
Theileria ovis isolate SH. T5	MN704656.1	99.81	99	0.0	Sheep	Iraq
Theileria ovis isolate THOD2	MN625903.1	99.81	99	0.0	Donkey	Egypt
Theileria ovis isolate THOB2	MN625887.1	99.81	99	0.0	Buffalo	Egypt
Theileria ovis isolate THOSH5	MN625886.1	99.81	99	0.0	Sheep	Egypt
Theileria ovis isolate SH.S2	MN544931.1	99.81	99	0.0	Sheep	Iraq
Theileria ovis isolate 3kz7	MN493111.1	99.81	99	0.0	Sheep	Turkey
Theileria sp. Iwate 141 gene	AB602888.1	99.81	99	0.0	*Cervus nippon*	Japan
Theileria sp. Iwate 276 gene	AB602887.1	99.81	99	0.0	*Cervus nippon*	Japan
Theileria sp. Iwate 228 gene	AB602886.1	99.81	99	0.0	*Cervus nippon*	Japan
Theileria sp. Iwate 194 gene	AB602885.1	99.81	99	0.0	*Cervus nippon*	Japan
Theileria sp. Iwate 169 gene	AB602883.1	99.81	99	0.0	*Cervus nippon*	Japan

Table S5. Prevalence of microbes in *Hyalomma* ticks in UAE.

**Sr. No.**	**Farm Locations**	**Samples (Camels)**	**Samples (Cows)**	**Samples (Sheep)**	**Samples (Goats)**	**Total Samples**	***Francisella* sp. (positive samples)**	***Rickettsia* sp. (positive samples)**	***Theileria annulata* (positive samples)**	***Theileria ovis* (positive samples)**
Abu Dhabi										
1.	Al-Foah, UAEU	2	-	-	-	2	1	-	-	-
2.	Beda Bent Saud	6	-	-	-	6	1	-	-	-
3.	Livestock Market, Al Ain	11	-	4 (36)	-	15	1	-	-	-
4.	Nahel Town	24	-	-	-	24	2	2	-	-
5.	Omghafa	84	-	-	-	84	3	1	-	-
6.	Truck Road	20	-	-	-	20	4	-	-	-
7.	Al-Wagan	30	-	-	-	30	5	1	-	-
8.	Dubai Road	70	0 (15)	-	-	70	4	1	-	-
9.	Bukriya	15	-	-	-	15	5	2	-	-
10.	Al-Saad	53	-	-	-	53	4	-	-	-
11.	Al-Hayer	5	-	-	-	5	-	-	-	-
12.	Swehan	24	-	-	-	24	-	-	-	-
13.	Nabagh	15	-	-	-	15	-	-	-	-
14.	Al-Dahra	50	-	-	0 (30)	50	-	-	-	-
15.	Al-Yahar	6	-	-	-	6	-	-	-	-
16.	Malaket	12	-	-	-	12	-	-	-	-
17.	Remah	5	-	-	-	5	-	-	-	-
18.	Beda Fares	17	-	-	-	17	-	-	-	-
19.	Seh Saba	3	-	-	-	3	-	-	-	-
20.	Bilayat	2	-	-	-	2	-	-	-	-
21.	Al-Arad	44	-	-	-	44	-	-	-	-
Sub-total		498 (498)	0	4 (36)	0	502 (534)	30	7		
Dubai										
22.	Dubai Camel Hospital	17 (17)	-	-	-	17	-	-	-	-
23.	Al-Qusais Market, Dubai	-	20 (20)	9 (9)	9 (9)	38	-	-	-	-
Sub-total		17 (17)	20 (20)	9 (9)	9 (9)	55 (55)				
Sharjah										
24.	Kalba, Sharjah	1 (3)	2 (6)	1 (25)	1 (25)	5	-	-	1	1
Sub-total		1 (3)	2 (6)	1 (25)	1 (25)	5 (59)	-	-	1 (detected in ticks from cows)	1 (detected in ticks from goats)
Total		516 (518)	22 (26)	14 (70)	10 (34)	562 (648)	30	7	1	1

Numbers in parenthesis represent the number of animals.
